# Sphingolipid Organization in the Plasma Membrane and the Mechanisms That Influence It

**DOI:** 10.3389/fcell.2016.00154

**Published:** 2017-01-10

**Authors:** Mary L. Kraft

**Affiliations:** Department of Chemical and Biomolecular Engineering, University of IllinoisUrbana, IL, USA

**Keywords:** sphingolipid distribution, plasma membrane organization, lipid domains, secondary ion mass spectrometry, SIMS, imaging

## Abstract

Sphingolipids are structural components in the plasma membranes of eukaryotic cells. Their metabolism produces bioactive signaling molecules that modulate fundamental cellular processes. The segregation of sphingolipids into distinct membrane domains is likely essential for cellular function. This review presents the early studies of sphingolipid distribution in the plasma membranes of mammalian cells that shaped the most popular current model of plasma membrane organization. The results of traditional imaging studies of sphingolipid distribution in stimulated and resting cells are described. These data are compared with recent results obtained with advanced imaging techniques, including super-resolution fluorescence detection and high-resolution secondary ion mass spectrometry (SIMS). Emphasis is placed on the new insight into the sphingolipid organization within the plasma membrane that has resulted from the direct imaging of stable isotope-labeled lipids in actual cell membranes with high-resolution SIMS. Super-resolution fluorescence techniques have recently revealed the biophysical behaviors of sphingolipids and the unhindered diffusion of cholesterol analogs in the membranes of living cells are ultimately in contrast to the prevailing hypothetical model of plasma membrane organization. High-resolution SIMS studies also conflicted with the prevailing hypothesis, showing sphingolipids are concentrated in micrometer-scale membrane domains, but cholesterol is evenly distributed within the plasma membrane. Reductions in cellular cholesterol decreased the number of sphingolipid domains in the plasma membrane, whereas disruption of the cytoskeleton eliminated them. In addition, hemagglutinin, a transmembrane protein that is thought to be a putative raft marker, did not cluster within sphingolipid-enriched regions in the plasma membrane. Thus, sphingolipid distribution in the plasma membrane is dependent on the cytoskeleton, but not on favorable interactions with cholesterol or hemagglutinin. The alternate views of plasma membrane organization suggested by these findings are discussed.

## Introduction

The plasma membranes of mammalian cells contain many different lipid species, but the distribution of sphingolipids within the plasma membrane and the mechanisms responsible for this organization are of particular interest. Sphingolipids function as structural components in cellular membranes, and they are metabolized to signaling molecules that modulate diverse cellular processes, ranging from apoptosis (Herr et al., 1997; Carpinteiro et al., 2008; Yabu et al., 2015) to cytoskeletal reorganization (Bartke and Hannun, 2009; Milhas et al., 2010; Gandy et al., 2013; Adada et al., 2014). Regulation of sphingolipid metabolite signaling likely involves segregating the parent sphingolipid molecules within distinct plasma membrane domains, but the distributions of various sphingolipids within the plasma membrane are not well established. At present, the different subspecies within the sphingolipid family are known to vary in terms of their chemical properties, expression patterns, specific protein binding partners, and consequently, specialized functions (Hannun and Bell, 1989; Mutoh et al., 1995; Snook et al., 2006; Yu et al., 2011; Contreras et al., 2012; Fantini and Yahi, 2015; Prasanna et al., 2016). These divergent properties and functions may suggest that each sphingolipid subspecies is compartmentalized within a different region of the plasma membrane. Nonetheless, most studies have focused on just a few types of sphingolipid-enriched plasma membrane domains: lipid rafts and ceramide-rich domains.

The lipid raft is likely the most intensely studied sphingolipid domain that hypothetically exists in the plasma membrane. Lipid rafts are defined as small (<200 nm) and dynamic plasma membrane domains that are enriched with cholesterol, sphingolipids, and glycosylphosphatidylinositol (GPI)-anchored proteins (Pike, 2006; Lingwood and Simons, 2010; Nyholm, 2015; Levental and Veatch, 2016). Favorable interactions between the cholesterol and sphingolipids are widely thought to drive lipid raft formation, producing higher ordering within this domain than in the surrounding membrane (Simons and Ikonen, 1997; Rietveld and Simons, 1998). GPI-anchored proteins and some transmembrane proteins are postulated to have an affinity for the distinct chemical and physical environment within the lipid raft, which hypothetically promotes their association with these domains and interactions between the proteins within them (Simons and Ikonen, 1997; Lingwood and Simons, 2010; Levental and Veatch, 2016). Protein-protein interactions are proposed to stabilize the small and dynamic rafts, leading to the formation of larger structures (Harder and Simons, 1999; Nyholm, 2015; Simons, 2016). Lipid rafts are hypothesized to mediate many important cellular processes, including protein trafficking, signal transduction, and virus budding (Scheiffele et al., 1999; Nguyen and Hildreth, 2000; Simons and Toomre, 2000; Schuck and Simons, 2004; Ono and Freed, 2005; Luo et al., 2008; Takahashi and Suzuki, 2011). The postulated higher ordering of the sphingolipids, cholesterol, and proteins within lipid rafts was thought to make these putative domains insoluble in cold ionic detergents (Schroeder et al., 1994; Ahmed et al., 1997; Cremesti et al., 2002; Zajchowski and Robbins, 2002). Consequently, detergent extraction was once widely used to study lipid rafts. Detergent-resistant membranes isolated from cells later proved to be artificial structures that were not present *in vivo* (Lichtenberg et al., 2005). This increased the importance of imaging putative raft components, such as sphingolipids and GPI-anchored proteins, within intact cell membranes.

Ceramide-rich domains in the plasma membrane have also been the subject of many studies. These domains are produced by the hydrolysis of sphingomyelin to ceramide by sphingomyelinase in response to stimuli (i.e., multivalent binding to membrane receptors; Cremesti et al., 2001; Bollinger et al., 2005). Like lipid rafts, ceramide-rich domains are postulated to exhibit high ordering that enhances the recruitment of GPI-anchored proteins, which modulates their interactions with other membrane proteins (Cremesti et al., 2002; Bollinger et al., 2005). However, ceramide-rich domains are large enough to be detected with light microscopy, and they putatively lack cholesterol enrichment (Cremesti et al., 2002; Bollinger et al., 2005). In this review, ceramide-rich domains are defined solely according to their enrichment with ceramide, irrespective of their cholesterol or protein content.

The following sections describe the sphingolipid distributions that have been imaged in resting cells with a variety of techniques, and how these organizations are affected by various stimuli. Due to space limitations, this review focuses on reports that contextualize the development of current models of plasma membrane organization, and the results that that have led some to question or even reject the raft hypothesis (Shaw, 2006; Kenworthy, 2008; Kraft, 2013; Sevcsik and Schütz, 2016; Wüstner et al., 2016). Emphasis is placed on the findings acquired with a new approach for chemically mapping isotope-labeled lipids in the plasma membrane with high-resolution, which were reported by the author and collaborators. Finally, the implications of these findings on models of sphingolipid organization in the plasma membrane are discussed.

## Methods to image sphingolipid distribution in the plasma membranes of mammalian cells

In order to visualize the sphingolipids within the plasma membrane, they must be functionalized with a label that can be detected with an imaging technique. A variety of lipid probes and detection methods have been employed, each having distinct advantages and disadvantages. One of the most common strategies to date is to use an affinity tag, such as an antibody or toxin, to label the sphingolipid species of interest. Noteworthy, non-toxic recombinant versions of toxin molecules that retain their sphingolipid-binding properties have been developed to permit live-cell imaging without adversely affecting cell viability (Kishimoto et al., 2016). The affinity tag is usually conjugated to a fluorophore or heavy metal particle that can be visualized with fluorescence or immunoelectron microscopy, respectively. Alternatively, the affinity tag is labeled with a second affinity tag (i.e., a polyclonal antibody) that has been functionalized to permit detection. This approach is attractive because it enables attaching any desired detection probe to endogenous lipids on the cell surface. The main limitation is that only a fraction of the lipid molecules of interest can typically be labeled and detected with an affinity label. This low detection efficiency is primarily due to three factors. First, affinity labels often cannot access the entire cell surface due to their relatively large size; second, lipids that are already bound to endogenous proteins cannot be detected; third, affinity label binding often depends on the specific orientation and/or clustering of the target lipid (Mahfoud et al., 2010; Mizuno et al., 2011; Kishimoto et al., 2016). Another disadvantage is that some anti-glycosphingolipid antibodies and the popular affinity label for GM1, cholera toxin subunit B, may also bind to glycoproteins, which compromises their ability to report the distribution of the target glycosphingolipid (Tonegawa and Hakomori, 1977; Blank et al., 2007; Day and Kenworthy, 2012; Wands et al., 2015).

The direct imaging of fluorophore-labeled sphingolipid analogs incorporated into the membranes of living cells has been gaining popularity. These fluorescent sphingolipid analogs are advantageous because they afford more flexibility in terms of fluorophore selection, and they can be employed for live cell imaging. Fluorescent sphingolipid precursors that permit observing the lipid distribution that results from biosynthesis and trafficking have also been developed (Peters et al., 2007; Kim et al., 2013). The main drawback to this approach is that the relatively large and chemically distinct fluorophore may alter the interactions between the labeled sphingolipid and other membrane components, which can change the lipid distribution in the membrane (Devaux et al., 2002; Maier et al., 2002; Shaw et al., 2006).

The sphingolipid distribution in the plasma membranes of intact cells has also been imaged with a high-resolution secondary ion mass spectrometry (SIMS) technique. High-resolution SIMS performed on a commercial instrument, the Cameca NanoSIMS 50, enables visualizing the distributions of metabolically incorporated stable isotope-labeled lipids in the plasma membranes of intact cells with better than 100 nm lateral resolution (Klitzing et al., 2013; Kraft and Klitzing, 2014). The principles of SIMS performed with a Cameca NanoSIMS 50 instrument have been previously described in detail (Boxer et al., 2009; Kraft and Klitzing, 2014). Therefore, the following description emphasizes the aspects of the technique that affect its application to imaging the lipid distribution in the plasma membranes of intact cells.

During NanoSIMS analysis, a cesium primary ion beam with a diameter of ~70 nm is raster scanned across the surface of the cell. The molecules within the beam's focal area are fragmented into small pieces, and the charged particles, which are called secondary ions, are ejected from the surface (top 5–10 nm) of the sample. This shallow depth of secondary ion ejection minimizes the detection of secondary ions from intracellular membranes, thereby restricting the analysis to the plasma membrane. The high-yielding monoatomic and diatomic secondary ions are collected by a mass spectrometer that can discriminate between ions that have the same nominal mass but different isotopic or elemental compositions (i.e., ^13^C^14^N^−^ at 27.0059 and ^12^C^15^N^−^ at 26.9996 amu). The intensities of the secondary ions detected at each pixel reveal the elemental and isotopic composition at the surface of the sample. Because elemental composition cannot be used to distinguish between lipid species, the sphingolipids must be labeled with distinct stable isotopes to allow their identification with a NanoSIMS instrument. This is achieved by metabolic labeling with isotope-labeled lipid precursors (Klitzing et al., 2013).

The strengths and weaknesses of high-resolution SIMS are complementary to those of imaging affinity tagged or fluorophore-labeled lipids with fluorescence microscopy. The strengths are that the stable isotope labels do not change the labeled lipid's chemical structure or molecular interactions, so its intracellular trafficking and distribution are not perturbed. Additionally, because distinct stable isotopes can be selectively and metabolically incorporated into the majority of the cellular sphingolipids, most sphingolipid molecules within the plasma membrane can be detected. The primary disadvantage is that this technique is performed under ultrahigh vacuum (UHV), so the cells must be dehydrated prior to analysis. However, previous studies demonstrate that chemical fixation techniques that crosslink the proteins with glutaraldehyde and the lipids with osmium tetroxide (OsO_4_) preserve the laminar structure of biological membranes and prevent lipid reorganization during sample dehydration and subsequent analysis (Stoechenius et al., 1960; Frisz et al., 2013b). Consequently, the NanoSIMS images acquired from chemically fixed cells represent snapshots of the lipid organizations that were present in the moments prior to fixation.

The following sections summarize some of the results that have been acquired with the aforementioned approaches. Studies that used fluorescence or immunoelectron microscopy to detect sphingolipid-specific affinity tags to probe the involvement of a specific type of sphingolipid domain in cell response to external stimuli (i.e., involvement of lipid rafts or ceramide-rich domains in receptor clustering) are presented first. Next, studies that employed affinity-labeled sphingolipids and fluorescent sphingolipid analogs to visualize the distributions of specific sphingolipid subspecies in the plasma membranes of unstimulated cells are described. This includes a brief account of the insights into plasma membrane organization that were acquired with super-resolution fluorescence techniques. Then the sphingolipid distributions that have been imaged in the plasma membranes of intact mammalian cells with high-resolution SIMS are summarized. Finally, the implications of these experimental results on our view of plasma membrane organization are discussed.

## Glycosphingolipid redistribution induced by antigen crosslinking

Antibody binding to proteins on the surfaces of lymphocytes was first reported to induce the crosslinked proteins to form clusters that eventually segregate into a large patch, or “cap” at one end of the cell in 1971 (Taylor et al., 1971). Subsequent reports showed this capping is inhibited by drug treatments that impair microtubules (De Petris, 1974), and it can be induced on any motile mammalian cell by crosslinking its surface antigens with multivalent ligands, such as antibodies (Bretscher, 1984). An early hypothesis for the crosslinking-induced capping of membrane proteins postulated that cell surface proteins are associated with cytoskeletal components that actively cluster the crosslinked membrane proteins in response to multivalent binding interactions (de Petris, 1977). This hypothesis predicts that crosslinking the glycosphingolipids that reside in the outer leaflet of the plasma membrane would not induce capping because these glycosphingolipids are not in direct contact with the cytoskeletal components in the cytoplasm. This prediction motivated the earliest efforts to characterize sphingolipid distribution in the plasma membrane in response to antigen capping. In 1975, Revesz and Greaves tested the prediction by labeling the GM1 in the plasma membranes of immune cells with cholera toxin, crosslinking the toxin with horse anti-cholera serum, and then labeling with fluorescent anti-horse secondary antibodies for visualization. They found the fluorescently labeled and crosslinked GM1 redistributed into multimicrometer-scale caps on the surfaces of the immune cells (Revesz and Greaves, 1975). The same year, Craig and Cuatrecasas reported that solely the binding of fluorescently labeled cholera toxin to GM1 was sufficient to induce the formation of large GM1 clusters on the surfaces of rat lymphocytes (Craig and Cuatrecasas, 1975). Like the capping of proteinaceous antigens, GM1 capping was inhibited by metabolic poisons and drugs that inhibit microtubules and microfilaments (Craig and Cuatrecasas, 1975; Revesz and Greaves, 1975). The sensitivity to microtubule and microfilament inhibitors implied that GM1 capping was mediated by cytoskeletal components. This unexpected implication instigated concerns that cholera toxin crosslinks both GM1 and glycosylated membrane proteins, and the observed capping was orchestrated by the cytoskeletal components associated with the crosslinked membrane glycoproteins.

Subsequent studies confirmed that capping could be induced by crosslinking glycosphingolipids with multivalent ligands other than cholera toxin. Exogenous Forssman glycolipid, a neutral glycosphingolipid consisting of five monosaccharides, inserted into mouse thymocytes could be capped by labeling it with a monoclonal primary antibody and then crosslinking with secondary antibodies (Stern and Bretscher, 1979). This capping was inhibited by chemically fixing the cells prior to crosslinking with the secondary antibody, and consistent with prior reports, by treatment with metabolic poisons or inhibitors of microfilaments and microtubules (Stern and Bretscher, 1979). Antibody crosslinking of the Forssman glycosphingolipid and globoside, a neutral glycosphingolipid with four monosaccharides, induced their aggregation in the membranes of erythrocytes (Tillack et al., 1983). However, anti-glycosphingolipid antibodies were reported to have an affinity for glycoproteins (Tonegawa and Hakomori, 1977), so these findings did not dissuade concerns that the observed capping was actually induced by the crosslinking of cell surface glycoproteins.

Spiegel and coworkers performed similar studies using gangliosides functionalized with non-native haptens (i.e., fluorophores) or biotin that could be crosslinked with antibodies or avidin, respectively, to ensure that the glycosphingolipid crosslinker had no affinity for endogenous proteins. The crosslinking of these exogenously incorporated gangliosides in the membranes of lynphocytes induced the formation of large patches and caps (Spiegel et al., 1979, 1984; Spiegel and Wilchek, 1981). Interestingly, anti-rhodamine antibodies elicited the co-aggregation of both rhodamine-labeled gangliosides and Lucifer yellow-labeled gangliosides on lymphocytes that contained both labeled gangliosides. However, anti-rhodamine antibodies did not induce the capping of Lucifer yellow-labeled gangliosides on lymphocytes that lacked rhodamine-labeled gangliosides (Spiegel et al., 1984). These experiments clearly demonstrate that capping can be induced by the crosslinking of glycosphingolipids, and also suggest that different gangliosides interact with one another within the plasma membrane.

The finding that metabolic poisons and inhibitors of cytoskeletal components impede glycosphingolipid capping (Craig and Cuatrecasas, 1975; Revesz and Greaves, 1975; Stern and Bretscher, 1979) implies that this capping involves energy-dependent cytoskeletal reorganization. But how could glycosphingolipid capping be mediated by cytoskeletal reorganization if the crosslinked glycosphingolipids on the cell surface do not contact the cytoplasm where cytoskeletal proteins reside? One hypothesis proposed that the glycosphingolipids selectively bind to membrane proteins that are associated with cytoskeletal components, and ligand binding induces cytoskeletal reorganization that actively clusters the crosslinked glycosphingolipids (Craig and Cuatrecasas, 1975; Bourguignon and Singer, 1977; Kellie et al., 1983). By the early 1980s, several reported observations indirectly supported this hypothetical model for glycosphingolipid capping. They included the detection of glycosphingolipids in isolated membrane protein complexes (Ji, 1974; Lingwood et al., 1980), the association of GM1 with cytoskeletons produced by detergent treatment (Sahyoun et al., 1981; Streuli et al., 1981; Hagmann and Fishman, 1982), and the accumulation of cytoskeletal proteins under the patches of crosslinked glycosphingolipids in intact cells (Kellie et al., 1983). An alternative hypothetical mechanism for ganglioside capping proposed that gangliosides self-associate with one another in resting cells, and crosslinking pulls these tiny ganglioside clusters together, forming larger lipid patches (Spiegel et al., 1984; Thomas et al., 1994). This hypothesis is consistent with the finding that GM1 crosslinking induced the co-capping of both GM1 and GM3 (Spiegel et al., 1984). However, this hypothetical mechanism for crosslinking-induced ganglioside capping did not predict a role for cytoskeletal components, or consequently, the impairment of ganglioside capping by metabolic poisons and inhibitors of cytoskeletal components.

The idea that lipid self-association drives the formation of distinct lipid domains that mediate capping and subsequent signal transduction further developed into the lipid raft hypothesis. This hypothesis states that attractive forces between sphingolipid and cholesterol molecules within the plasma membrane give rise to ordered cholesterol- and sphingolipid-enriched domains that are called lipid rafts (Simons and Ikonen, 1997). GPI-anchored proteins are hypothesized to have an affinity for, and thus concentrate within lipid rafts, thereby promoting their interactions with other raft-associated signaling proteins (Simons and Ikonen, 1997). The presence of lipid rafts at the site of antigen patching was inferred from the co-patching of crosslinked receptors and gangliosides, which are purportedly integral lipid raft components, on the surfaces of immune cells (Stauffer and Meyer, 1997; Harder et al., 1998). Clusters of GPI-anchored receptors and gangliosides were not detected on cells without crosslinking (Mayor et al., 1994; Mayor and Maxfield, 1995; Fujimoto, 1996). Therefore, GPI-anchored proteins were hypothesized to reside in tiny lipid rafts that nucleate into structures that can be detected with conventional fluorescence microscopy when crosslinked (Harder et al., 1998). Actin accumulated under the crosslinked antigen patches, so these larger protein clusters were hypothesized to represent the coalescence of lipid rafts into larger domains that were stabilized by the actin cytoskeleton and its associated proteins (Ash et al., 1977; Bourguignon and Singer, 1977; Kellie et al., 1983; Pierini et al., 1996; Harder and Simons, 1999). The hypothesis that lipid raft clustering is responsible for the patching of crosslinked antigens was bolstered by the early finding that the co-clustering of crosslinked GPI-anchored proteins and GM1 was reduced by cholesterol depletion, which ostensibly eliminates lipid rafts (Harder et al., 1998; Harder and Simons, 1999).

The hypothetical role of lipid rafts in antigen patching stimulated new efforts to image the glycosphingolipid reorganization induced by antigen crosslinking. Based on the assumptions that GM1 and other gangliosides are markers for lipid rafts, and favorable cholesterol-sphingolipid interactions drive lipid raft formation, many studies focused on imaging GM1 proximity to crosslinked antigens and the effects of cholesterol depletion. These studies confirmed that antigen crosslinking induces local elevations in the fluorescence signals from both the crosslinked GPI-anchored protein and toxin-crosslinked GM1, and this co-clustering is inhibited by cholesterol depletion (Stauffer and Meyer, 1997; Harder et al., 1998; Huby et al., 1999; Janes et al., 1999; Grassmé et al., 2001a; Mitchell et al., 2002). Subsequent reports also confirmed that cytoskeletal elements accumulate under the site of antigen patching (Rodgers and Zavzavadjian, 2001; Delaguillaumie et al., 2004; Wilson et al., 2004).

Though many reports verified the signals from GM1 and the clustered membrane proteins were colocalized at the resolution of conventional fluorescence microscopy, other reports challenged the interpretation of this co-localization as evidence for antigen clustering in rafts. Fluorescence resonance energy transfer (FRET) studies indicated a lack of true co-localization between GPI-anchored proteins and cholera toxin-labeled GM1 (Kenworthy et al., 2000; Glebov and Nichols, 2004). The energy transfer between the antibody-labeled GPI-anchored proteins and cholera toxin B-labeled GM1 correlated with their surface densities, and were not selectively colocalized, which is inconsistent with GPI-anchored protein recruitment to lipid rafts (Kenworthy et al., 2000; Glebov and Nichols, 2004). The local increases in fluorescence from GPI-anchored proteins and cholera toxin-labeled GM1 observed after antigen crosslinking could instead be attributed to a local excess of cell membrane. Consistent with this conclusion, another report clearly showed numerous membrane folds and protrusions were present at the site where the fluorescence signals from the GPI-anchored proteins and cholera toxin-labeled GM1 were elevated on a Jurkat cell (Glebov and Nichols, 2004). An immunoelectron microscopy study also challenged the finding that crosslinked GPI-anchored proteins co-cluster with GM1. This work revealed a lack of GM1 enrichment in patches of crosslinked putative raft proteins, namely the GPI-anchored protein Thy-1 and the IgE receptor (Wilson et al., 2004), which argues that these crosslinked antigens do not reside in lipid rafts. Consequently, the observed patching of Thy-1 and IgE receptor could not have been mediated by either lipid rafts or the favorable cholesterol-sphingolipid interactions that hypothetically drive raft formation.

Recent reports that cholesterol depletion perturbs cytoskeletal organization (Ramprasad et al., 2007; Sun et al., 2007; Qi et al., 2009; Norman et al., 2010; Chubinskiy-Nadezhdin et al., 2013; Dick et al., 2013) may suggest that cholesterol depletion inhibits antigen patching by preventing the cytoskeletal proteins from actively clustering the crosslinked antigens. But, as mentioned above, if the cytoskeleton, and not lipid rafts, mediates the clustering of crosslinked antigens, the finding that crosslinking induces glycosphingolipid capping implies the glycosphingolipids in the outer leaflet of the plasma membrane are indirectly associated with cytoskeletal proteins. Studies of the trafficking of GD3, a disialoganglioside ganglioside, during CD95/Fas-mediated apoptosis seem to support this possibility. CD95/Fas-mediated apoptosis is initiated by the binding of either the Fas ligand or an antagonistic Fas antibody to CD95, a member of the TNF-receptor superfamily that is also called Fas (Wajant, 2014). This binding induces the recruitment of Fas-associated death domain (FADD) to the CD95 death domain. Next, procaspase-8 is recruited to FADD's death effector domain, forming the death-inducing signaling complex (DISC) that elicits apoptosis (Algeciras-Schimnich et al., 2002; Wajant, 2014). Interest in GD3 involvement in CD95/Fas-mediated apoptosis began with the discovery that the crosslinking of CD95 on lymphoid and myeloid cells induces GD3 production, and this ganglioside is required for apoptosis (De Maria et al., 1997). Immunoelectron and immunofluorescence imaging of GD3 and organelle markers in hepatocytes treated with tumor necrosis factor-α (TNF-α) revealed that GD3 moved from the plasma membrane to mitochondria prior to mitochondrial membrane depolarization and apoptosis (Garcıa-Ruiz et al., 2002). Malorni and coworkers identified multiple cytoskeletal proteins that GD3 may associate with during its transit to mitochondria in lymphoid cells treated with anti-CD95 antibodies. GD3 association with ezrin was suggested by the co-localization between ezrin and GD3 observed with immunofluorescence microscopy, and by the presence of GD3 in immunoprecipitates obtained with anti-ezrin monoclonal antibodies (Giammarioli et al., 2001). Another study by Malorni and coworkers provided strong evidence that GD3 also associates with tubulin (Sorice et al., 2009). This evidence includes the elevated FRET efficiency between GD3 and β-tubulin that was detected after Fas ligation, immunoelectron images showing immunogold-labeled GD3 on microtubules, and the presence of GD3 in immunoprecipitates obtained with anti-tubulin antibodies (Sorice et al., 2009). Furthermore, an *in silico* docking analysis predicted GD3 has a high affinity for a pore on polymerized tubulin, indicating selective GD3-tubulin interactions (Sorice et al., 2009). A subsequent FRET study revealed that GD3 colocalized with CLIPR-59, a tubulin-binding protein, shortly before it colocalized with tubulin (Sorice et al., 2010). Based on the assumption that GD3 is a marker for lipid rafts, it had been proposed that GD3 trafficking involved interactions between lipid rafts and the cytoskeleton (Giammarioli et al., 2001; Sorice et al., 2009, 2010). However, these results also support an alternative hypothesis that GD3 trafficking is mediated by the selective binding of individual GD3 molecules directly to proteins associated with the cytoskeleton in absence of lipid rafts.

Overall, the results described in this section clearly demonstrate that the crosslinking of glycosphingolipids induces their redistribution into patches on the surfaces of immune cells. However, they fail to conclusively establish whether either favorable interactions between cholesterol and sphingolipids or specific glycosphingolipid-protein interactions are the driving force for this glycosphingolipid reorganization.

## Ceramide-rich membrane domains induced by external stimuli

Ceramide's role as a second messenger that directly participates in signaling cascades began to gain recognition in the early 1990's (Kim et al., 1991; Dobrowsky and Hannun, 1992; Bielawska et al., 1993; Dobrowsky et al., 1993; Obeid et al., 1993; Cifone et al., 1994; Hannun, 1994). By the late 1990's, various stimuli were known to activate sphingomyelinases that hydrolyze sphingomyelin to ceramide, producing a transient increase in ceramide levels that is required for biological response (Wiegmann et al., 1994; Tepper et al., 1995; Grassmé et al., 1997; Brenner et al., 1998; Junge et al., 1999; Grullich et al., 2000). This section describes studies that probed the subcellular localization of sphingomyelinase and the ceramide it produces in response to external stimuli.

Among the stimuli that induce ceramide generation is the crosslinking of CD95 (Cifone et al., 1994; Tepper et al., 1995; Brenner et al., 1998; Grullich et al., 2000), which also induces GD3 production and its trafficking within the cell (*vide supra*). Immunoimaging studies established that CD95 activation induces acid sphingomyelinase translocation to the cell surface and subsequent CD95 clustering (Grassmé et al., 2001a; Lacour et al., 2004). Ceramide generation in the plasma membrane was initially postulated to occur in caveolae, which are flask-shaped plasma membrane invaginations that consist of the caveolin-1 structural protein (Liu and Anderson, 1995; Bilderback et al., 1997). This hypothesis was based on the finding that sphingomyelin levels decreased and ceramide levels increased in a caveolin-rich detergent-insoluble membrane fraction that could be isolated from cells (Liu and Anderson, 1995; Bilderback et al., 1997). The caveolin-containing detergent insoluble membrane fraction was also enriched with cholesterol, sphingolipids, and GPI-anchored proteins, so after the raft hypothesis was proposed, ceramide generation was postulated to occur in lipid rafts (Grassmé et al., 2001a). Efforts to investigate this hypothesis often combined immunolabels for sphingomyelinase detection with the aforementioned strategies used to assess the involvement of lipid rafts in receptor clustering, such as imaging immunolabeled GM1 as a proxy for rafts and probing the effects of cholesterol depletion. These studies demonstrated that after translocation to the cell surface, the signals from the acid sphingomyelinase overlapped with those from the clustered CD95 and cholera toxin-labeled GM1 on the surfaces of CD95-activated cells (Grassmé et al., 2001a; Bock et al., 2003). Depletion of cellular cholesterol reduced acid sphingomyelinase translocation to the cell surface, subsequent CD95 clustering, and CD95-induced apoptosis (Cremesti et al., 2001; Grassmé et al., 2001a; Lacour et al., 2004). The authors concluded that acid sphingomyelinase is transported to lipid rafts where it generates the ceramide that is required for receptor clustering and subsequent apoptosis. Noteworthy, this conclusion hinges on the assumptions that GM1 primarily resides in lipid rafts, and that cholesterol depletion eliminates lipid rafts without perturbing specific protein-protein or cholesterol-protein interactions.

The use of new ceramide-specific affinity labels to study the role of ceramide generation in receptor clustering yielded compelling evidence for the existence of ceramide-rich domains in the plasma membrane (Grassmé et al., 2001b, 2002; Bock et al., 2003; Lacour et al., 2004). Immunofluorescence imaging of a fluorescently labeled protein construct with an affinity for ceramide revealed large fluorescent patches at the perimeters of CD95-stimulated Jurkat cells (Grassmé et al., 2001b). CD95 clustering was inhibited by treating the cells with proteins that bind to the ceramide on the cell surface prior to CD95 activation, and by inhibition of acid sphingomyelinase, which confirms ceramide generation is required for biological response (Grassmé et al., 2001b). A subsequent report that employed anti-ceramide antibodies to detect ceramide also indicated the presence of large ceramide-rich patches on CD95-activated colon cancer cells that had been treated with the anticancer drug cisplatin (Lacour et al., 2004). Overlap between the large patches of ceramide-specific fluorescence and the clustered CD95 at the cell periphery was detected with immunofluorescence imaging; neither patches of ceramide-specific fluorescence nor CD95 clusters were found on untreated cells (Lacour et al., 2004). The possibility that the elevated patches of fluorescence from the ceramide-specific affinity labels detected in these studies may signify an excess of membrane caused by membrane folds and protrusions has not been directly assessed. However, electron microscopy images of intact and sectioned cells demonstrated that acid sphingomyelinase was localized within distinct regions on the surfaces of CD95-activated cells, and was not evenly distributed on their surfaces (Grassmé et al., 2001a,b). Because the production of ceramide on the cell surface is catalyzed by acid sphingomyelinase, this compartmentalized acid sphingomyelinase distribution indicates ceramide is produced at discrete regions on the cell surface. Consequently, the elevated patches of ceramide-specific fluorescence observed in the studies described above likely represent ceramide-enriched membrane domains, and not an excess of membrane.

Subsequent studies involving the imaging of immunolabeled ceramide show that many stimuli, including the activation of other immune cell receptors, induce the acid sphingomyelinase-mediated formation of ceramide-rich domains (Grassmé et al., 2002; Abdel Shakor et al., 2004; Korzeniowski et al., 2007). The activation of cluster of differentiation 40 (CD40), a member of the TNF-receptor superfamily found on antigen presenting cells, induced the formation of ceramide patches that largely colocalized with clustered CD40 and acid sphingomyelinase (Grassmé et al., 2002). Similar to CD95, CD40 clustering, and subsequent signaling was inhibited by a loss of acid sphingomyelinase activity, neutralization of cell surface ceramide, and cholesterol depletion (Grassmé et al., 2002). Likewise, immunofluorescence imaging of ceramide showed the activation of Fc gamma receptor II (FcγRII), an immune cell receptor for IgG, induced acid sphingomyelinase activity at the cell surface and the formation of ceramide-rich membrane patches (Abdel Shakor et al., 2004; Korzeniowski et al., 2007). This ceramide production was required for the clustering of the crosslinked FcγRII, subsequent receptor phosphorylation, and signaling.

Some stimuli that ultimately trigger membrane internalization also induce acid sphingomyelinase translocation to the cell surface and the subsequent formation of ceramide-rich plasma membrane domains. This includes the internalization of pathogenic bacteria, viruses, cell-penetrating peptides, and nanoparticles functionalized with anti-intercellular adhesion molecule-1 (ICAM) antibodies (Grassmé et al., 1997, 2003a; Grassmé, 2005; Verdurmen et al., 2010; Serrano et al., 2012). Additionally, the binding of iron-loaded transferrin to the transferrin receptor results in the formation of ceramide-rich patches that are required for the recruitment transferrin/transferrin receptor complexes to clathrin-coated pits and their successive internalization (Abdel Shakor et al., 2012).

In the majority of these studies, the biological effects of ceramide production were hypothesized to involve changes in lipid-lipid interactions resulting from the hydrolysis of sphingomyelin in lipid rafts to ceramide. Cleavage of the phosphatidylcholine head group from sphingomyelin reduces the affinity between cholesterol and the newly formed ceramide (Megha and London, 2004). This hypothetically promotes a local loss of cholesterol and the formation of a ceramide-rich domain with a negative curvature that induces vesicle formation (Kolesnick et al., 2000; Cremesti et al., 2002; Megha and London, 2004; Bollinger et al., 2005). An alternative mechanism for ceramide-mediated receptor clustering and internalization invokes ceramide's role as a second messenger that mediates cytoskeletal remodeling and membrane internalization through selective ceramide-protein interactions. The ceramide produced in the plasma membrane by acid sphingomyelinase is known to selectively bind to and activate two protein phosphatases, PP2A and PP1 (Chalfant et al., 1999; Canals et al., 2010, 2012). These ceramide-activated serine/threonine phosphatases dephosphorylate ezrin, which abrogates the simultaneous binding of ezrin to actin and the plasma membrane, causing a loss of plasma membrane-cytoskeleton linkage, and cortical actin remodeling (Zeidan et al., 2008; Canals et al., 2010, 2012). Therefore, selective ceramide-protein interactions may mediate the cytoskeletal remodeling that is necessary for receptor clustering, internalization, and transport through the cortical actin network beneath the plasma membrane.

## Immunoimaging multiple sphingolipid species in parallel within the plasma membrane

The development of antibodies and non-toxic recombinant versions of toxin molecules that selectively bind to distinct sphingolipid subspecies has enabled simultaneously visualizing the distributions of multiple sphingolipid subspecies within the plasma membrane. Studies that imaged these new sphingolipid-specific affinity labels suggest that different sphingolipid subspecies are segregated within different regions of the plasma membrane (Fujita et al., 2007, 2009; Janich and Corbeil, 2007; Chen et al., 2008). One study probed the distributions of GM1, GM3, and prominin-1, a cholesterol-binding protein that resides in plasma membrane protrusions (Roper et al., 2000), on the apical surfaces of MDCK cells (Janich and Corbeil, 2007). This work showed that fluorescent cholera toxin B-labeled GM1 colocalized with antibody-labeled prominim-1 on microvilli on the apical surfaces of MDCK cells, whereas fluorescent antibody-labeled GM3 was excluded from these sites (Janich and Corbeil, 2007). In contrast, both fluorescent cholera toxin B-labeled GM1 and imunolabeled GM3 colocalized with the labeled prominin-1 on primary cilium, which are another type of protrusion on the apical surfaces of MDCK cells. A study that used near-field scanning optical microscopy (NSOM) and quantum dot-functionalized affinity labels to detect GM1 and GM3 on separate MDCK cells also indicated GM1 and GM3 were segregated on the apical cell surface (Chen et al., 2008). In this study, the GM3 and GM1 were primarily found on the peaks and valleys, respectively, of the microvillus-like protrusion on the apical surface of the MDCK cells (Chen et al., 2008).

A lack of co-localization between GM1 and GM3 on mouse fibroblast cells was also reported by Fujimoto and coworkers. They performed immunoelectron microscopy on flash-frozen and freeze-fractured mouse fibroblast cells that had been immunolabeled for GM1 and GM3 using orthogonal antibody pairs functionalized with different diameter colloidal gold particles (Fujita et al., 2007, 2009). Both GM3 and GM1 were clustered within separate plasma membrane domains that rarely overlapped. Cholesterol depletion reduced the abundances of the GM1 and GM3 clusters, which is consistent with the hypothesis that these gangliosides reside in rafts that are dependent on cohesive cholesterol-sphingolipid interactions (Fujita et al., 2007). However, chilling the cells on ice prior to flash-freezing, which was expected to promote the growth of the ordered lipid raft domains, actually reduced the clustering of GM1 and GM3 within the plasma membrane (Fujita et al., 2007). Interestingly, depolymerization of cellular actin by treatment with latrunculin A reduced the number of non-overlapping GM1 and GM3 domains in the plasma membrane, and increased GM1 and GM3 co-clustering (Fujita et al., 2009). Inhibition of Src-family kinases decreased the clustering of GM3 more significantly than GM1 (Fujita et al., 2009). The authors proposed that GM1 and GM3 might bind to different transmembrane proteins that associate with the cytoskeleton, and these different ganglioside-protein-cytoskeleton interactions are differentially influenced by cholesterol depletion and Src-family kinase inhibition.

Altogether, the simultaneous imaging of multiple immunolabeled ganglioside species points to the existence of multiple types of sphingolipid domains in the plasma membrane. These studies indicate that the mechanism for plasma membrane organization is far more complex than one governed by the components' differential affinities for ordered domains that are induced by cohesive cholesterol-sphingolipid interactions.

## Imaging fluorophore-labeled sphingolipids within the plasma membrane

The presence of multiple different types of sphingolipid domains within the plasma membrane was also suggested by studies that probed the distributions of various fluorescent sphingolipid analogs on the surfaces of mammalian cells. In these experiments, fluorophore-labeled sphingolipid analogs are incorporated into the plasma membranes of living cells and imaged with fluorescence microscopy. A complication of this approach is that the fluorescent lipid analogs can be internalized and incorporated into intracellular membranes. Labeled intracellular membranes, such as endosomes or vesicles, adjacent to the plasma membrane produce regions of elevated fluorescence that are difficult to discriminate from fluorescent membrane patches that signify a local enrichment in the fluorescent lipid. To avoid this complication, Tyteca and coworkers probed the distribution of fluorescent sphingolipid analogs in erythrocytes (Tyteca et al., 2010; D'Auria et al., 2013), which lack nuclei, endosomes, endoplasmic reticulum, and other membrane-bound organelles, and are also incapable of lipid metabolism and membrane trafficking. They used BODIPY-labeled analogs of sphingomyelin, glucosylceramide (BODIPY-GlcCer), and lactosylceramide (BODIPY-LacCer) in which the BODIPY fluorophore was attached to the N-acyl fatty acid. All three of these BODIPY-labeled sphingolipid analogs formed micron-sized domains in the plasma membranes of erythrocytes. Similar domains were observed when other fluorophores were used in place of BODIPY, which indicates this sphingolipid clustering was not induced by the fluorophore (Tyteca et al., 2010). A series of control experiments argued that the regions of elevated BODIPY-sphingolipid fluorescence on the erythrocytes signify plasma membrane domains enriched with BODIPY-sphingolipids, and not membrane folds or protrusions. Interestingly, the abundances of these BODIPY-sphingolipid domains did not progressively increase as temperature decreased (Tyteca et al., 2010), which argues against a phase separation-like process.

Membrane domains enriched with BODIPY-sphingomyelin, BODIPY-GlcCer, and BODIPY-LacCer were also detected on nucleated cells. Compared to erythrocytes, the sphingolipid-enriched domains appeared to be more abundant and elongated on Chinese hamster ovary (CHO) cells (Tyteca et al., 2010). Control experiments argued against the possibilities that these fluorescent patches were caused by the detection of excess membrane or the nonspecific absorption of aggregated BODIPY-sphingolipid analogs. Double labeling experiments revealed the BODIPY-sphingomyelin and BODIPY-LacCer formed separate domains in the plasma membranes of CHO cells, whereas BODIPY-GlcCer and BODIPY-LacCer colocalized within the same domains (Tyteca et al., 2010). Additionally, a GPI-anchored green fluorescent protein (GFP) construct colocalized with the BODIPY-LacCer domains, but not the BODIPY-sphingomyelin domains. The BODIPY-sphingomyelin domains were not affected by latrunculin A-induced actin depolymerization, but they coalesced into larger structures following depletion of ATP or 70% of the cholesterol in CHO cells (Tyteca et al., 2010).

In a subsequent report, Tyteca and coworkers reported BODIPY-labeled analogs of GM1 (BODIPY-GM1) and phosphatidylcholine (BODIPY-PC) also formed micron-scale domains in the plasma membranes of erythrocytes (D'Auria et al., 2013). The mechanism for BODIPY-PC domain formation was not clear. The abundances of the membrane domains enriched with BODIPY-GM1, BODIPY-PC, BODIPY-sphingomyelin, and BODIPY-GlcCer decreased when membrane tension increased due to cell spreading (D'Auria et al., 2013). Cholesterol depletion had little effect on the BODIPY-GlcCer domains on erythrocytes. However, cholesterol depletion eliminated the BODIPY-sphingomyelin and BODIPY-PC domains (D'Auria et al., 2013) on erythrocytes, which seems to contrast with the prior finding that cholesterol depletion induced the formation of large BODIPY-sphingomyelin domains on CHO cells (Tyteca et al., 2010). The abundances of BODIPY-GlcCer and BODIPY-sphingomyelin domains on the erythrocytes increased when the membrane-spectrin linkage was uncoupled, and proteins involved in membrane-spectrin anchorage colocalized with the BODIPY-sphingomyelin domains (D'Auria et al., 2013). Overall, the lack of colocalization between the different sphingolipid domains, their dependency on membrane-cytoskeleton anchorage, and the differential effects of cholesterol depletion on these domains are inconsistent with hypothetical mechanisms of sphingolipid domain formation driven solely by cohesive cholesterol-sphingolipid interactions. The authors proposed that the differential sensitivity of the various sphingolipid domains to cholesterol abundance may indicate regulation of membrane-cytoskeleton anchorage by cholesterol (D'Auria et al., 2013). Consistent with their hypothesis, the band 3 anion transport protein, which links the plasma membrane to the underlying cytoskeleton, reportedly has an affinity for cholesterol (Klappauf and Schubert, 1977; Schubert and Boss, 1982).

## Super-resolution fluorescence imaging of fluorescent sphingolipid analogs in the plasma membrane

The expectation that lipid rafts are too small and dynamic to be detected with diffraction-limited fluorescence microscopy motivated attempts to detect lipid rafts with super-resolution fluorescence microscopy techniques (Owen et al., 2012). Instead of imaging the sphingolipids and cholesterol in parallel at high spatial resolution, many studies focused on tracking the diffusion of fluorescent sphingolipid analogs or other putative raft components in the plasma membrane. The cohesive cholesterol- and sphingolipid interactions that hypothetically induce lipid raft formation would hinder the diffusion of these components in the plasma membrane, producing a detectable anomalous diffusion that would be sensitive to cholesterol depletion.

Perhaps the most influential super-resolution imaging studies of membrane organization revealed complex lipid dynamics that were ultimately inconsistent with partitioning into liquid-ordered membrane domains produced by favorable cholesterol-and sphingolipid interactions (Hiramoto-Yamaki et al., 2014; Honigmann et al., 2014; Andrade et al., 2015; Sevcsik et al., 2015). Stimulated emission depletion (STED) fluorescence microscopy imaging demonstrated fluorophore-labeled sphingomyelin, GM1, and a GPI-anchored protein were temporarily trapped within 20-nm-diameter areas in the plasma membrane of living cells, and this trapping was cholesterol-dependent (Eggeling et al., 2009). In comparison, identically labeled phosphatidylethanolamine appeared to diffuse freely in the membrane (Eggeling et al., 2009), which implied that lipid-cytoskeleton interactions were not responsible for the anomalous cholesterol-dependent sphingolipid diffusion. Noteworthy, this finding of unhindered phosphatidylethanolamine diffusion conflicts with a previous single molecule tracking study (Fujiwara et al., 2002), and subsequent STED-FCS and single molecule tracking studies reported by these authors and others (Andrade et al., 2015; Fujiwara et al., 2016; Komura et al., 2016). Although, the authors of the STED study never concluded that the cholesterol-dependent trapping of sphingomyelin, GM1 and GPI-anchored proteins was indicative of tiny lipid rafts, their results were often cited by others as support for the lipid raft hypothesis (Lingwood and Simons, 2010; Levental and Veatch, 2016). Subsequent studies showed that the transient trapping of the fluorescent sphingolipids and GPI-anchored proteins in the plasma membrane were both cholesterol- and cytoskeleton-dependent, and likely reflected binding to immobile membrane proteins, and not entrapment in lipid rafts (Mueller et al., 2011; Honigmann et al., 2014; Sevcsik et al., 2015). Super-resolution fluorescence microscopy imaging also revealed fluorescent cholesterol analogs diffuse freely in the plasma membranes of living cells (Hiramoto-Yamaki et al., 2014; Honigmann et al., 2014), which argues against the existence of lipid rafts.

## Direct imaging of sphingolipid distribution in the plasma membrane with high-resolution sims

High-resolution SIMS performed on a NanoSIMS 50 instrument was used to decisively answer the question: How are cholesterol and sphingolipids distributed in the plasma membranes of intact mouse fibroblast cells? Transfected NIH 3T3 mouse fibroblast cells that stably expressed influenza hemagglutinin (Clone 15 cell line) were employed in these experiments because the micrometer-scale hemagglutinin clusters in their plasma membranes were hypothesized to colocalize with lipid rafts (Scheiffele et al., 1997; Hess et al., 2005; Polozov et al., 2008). This hypothesis suggested that these cells had sphingolipid- and cholesterol-rich membrane domains that could easily be detected with high-resolution SIMS. Untransfected NIH 3T3 mouse fibroblast cells were also analyzed for comparison. Distinct stable isotopes, ^15^N and ^18^O, were metabolically incorporated into the sphingolipids and cholesterol, respectively, in living Clone 15 and NIH 3T3 cells (Klitzing et al., 2013). High levels of rare isotope incorporation into the cellular sphingolipids and cholesterol were achieved to ensure that the majority of the sphingolipid and cholesterol molecules in the plasma membrane could be detected and imaged with high-resolution SIMS.

**Figure 1:**
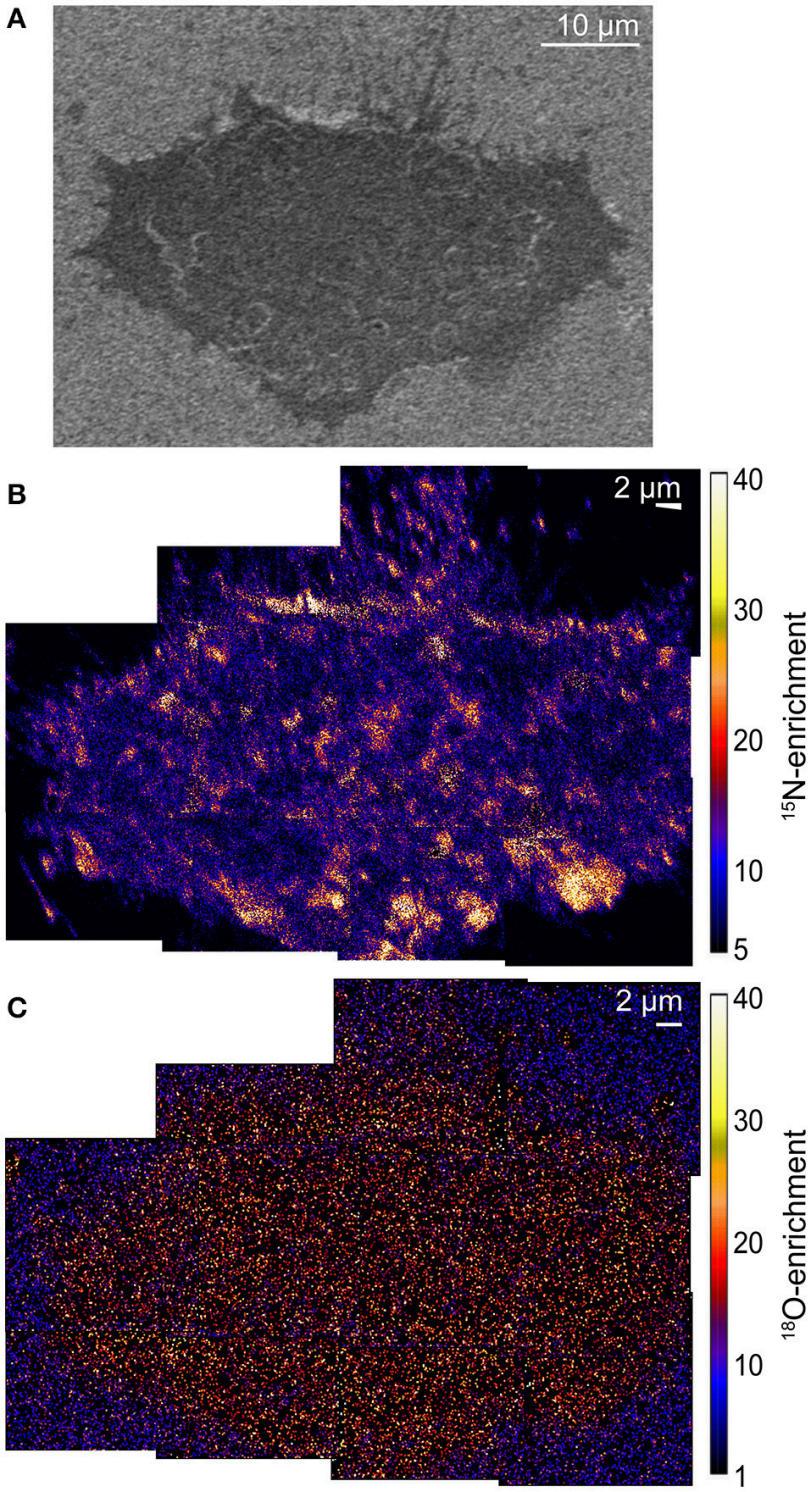
**SEM and SIMS images show the morphology of a NIH 3T3 mouse fibroblast cell and the sphingolipid and cholesterol distribution in its plasma membrane. (A)** SEM image of a NIH 3T3 fibroblast. **(B)** Montage of ^15^N-enrichment high-resolution SIMS images shows ^15^N-sphingolipid domains (orange and yellow regions) in the plasma membrane. **(C)** The ^18^O-enrichment images that were acquired in parallel show a relatively even ^18^O-cholesterol distribution in the plasma membrane. Color scales show the number of times that the ^15^N- or ^18^O-enrichment is greater than standard abundance. Montages consist of several high-resolution SIMS images that were acquired with 87-nm-lateral resolution. Adapted with permission from research originally published in Frisz et al. (2013a). © The American Society for Biochemistry and Molecular Biology.

The low-voltage SEM image (Figure 1A) shows the morphology of a representative chemically fixed NIH 3T3 mouse fibroblast cell (Frisz et al., 2013a). High-resolution SIMS imaging of the lipid-specific isotope enrichments on the cell showed the plasma membrane contained ^15^N-sphingolipid domains, evidenced by statistically significant local elevations in ^15^N-enrichment, that were as large as 2 μm across (Figure 1B; Frisz et al., 2013a,b). In contrast, ^18^O-cholesterol was uniformly distributed within the plasma membrane (Figure 1C) (Frisz et al., 2013a,b), and was not enriched at the sphingolipid domains (Frisz et al., 2013a). Similar sphingolipid and cholesterol distributions were observed on multiple other NIH 3T3 mouse fibroblast cells and Clone 15 cells (Frisz et al., 2013a,b).

**Figure 2:**
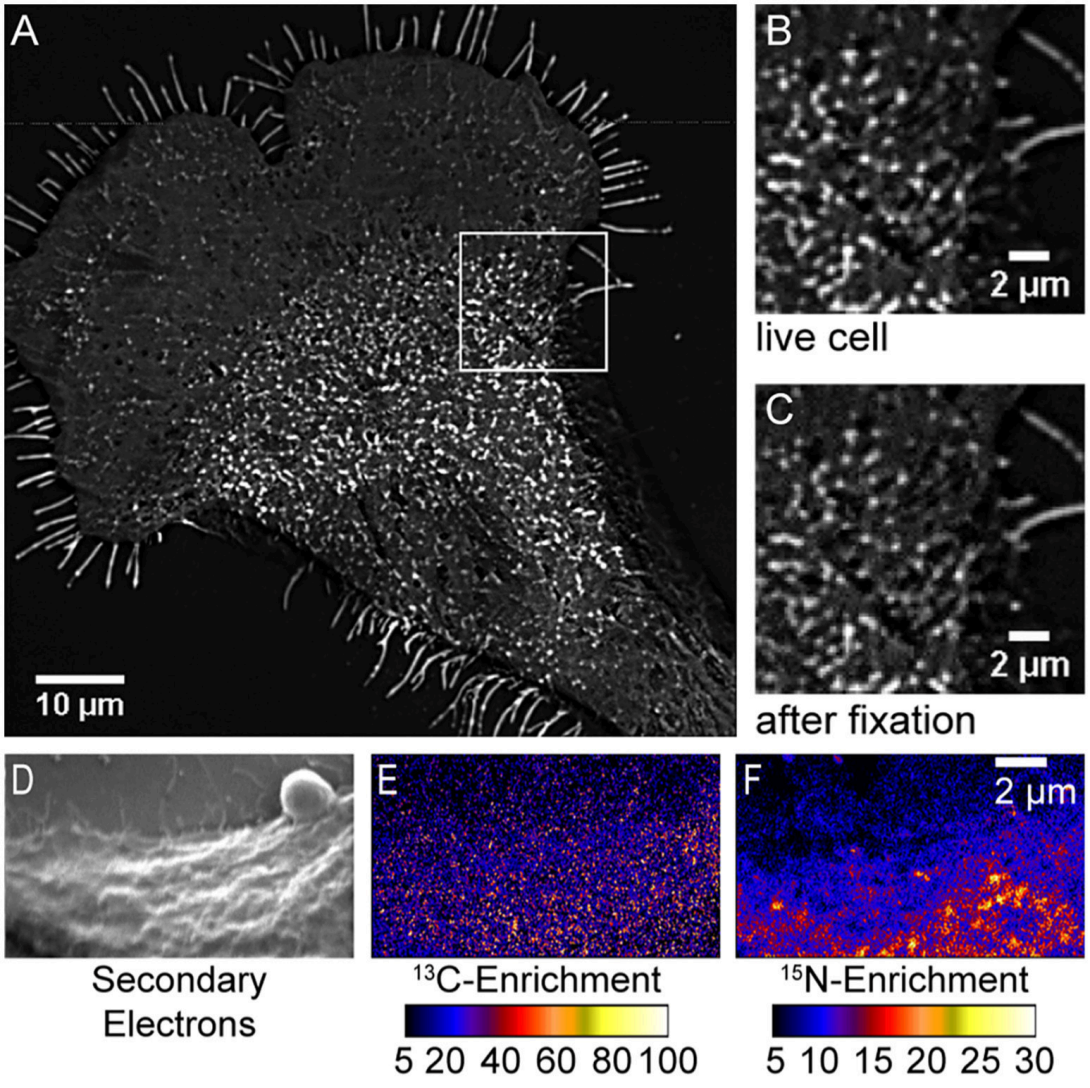
**Control experiments exclude possible artifacts caused by cell fixation or the detection of excess membrane caused by intracellular membranes adjacent to the plasma membrane**. Total internal reflectance microscopy images (background subtracted and averaged through the stack) of BODIPY-sphingolipids in the plasma membrane of a fibroblast **(A,B)** before and **(C)** after fixation. Enlargement of outlined region in **(A)** shows no change in the domains that were present **(B)** in the living cell **(C)** after glutaraldehyde fixation. Fluorescent micro-extensions are artifacts of background correction. Reproduced with permission from Frisz et al. (2013b). Copyright 2013 National Academy of Sciences, U.S.A. The **(D)** secondary electron, **(E)** ^13^C-enrichment, and **(F)** ^15^N-enrichment images acquired with high-resolution SIMS shows that the ^15^N-sphingolipid domains do not coincide with cell projections, folds, or other excesses of cellular lipids, which are labeled with carbon-13 and thus, would produce a co-elevation in ^13^C-enrichment. The color scale represents the indicated isotope enrichment measured at each pixel compared to unlabeled cells. Adapted with permission from Frisz et al. (2013b). Copyright 2013 National Academy of Sciences, U.S.A.

The finding of sphingolipid domains with dimensions sufficient for detection with fluorescence microscopy is consistent with the abovementioned reports of micron-scale domains of fluorescent sphingolipid analogs in the membranes of living cells (Tyteca et al., 2010; D'Auria et al., 2013). Though unexpected, the relatively uniform cholesterol distribution observed is consistent with previous reports that intrinsically fluorescent sterols are evenly distributed in the membranes of mammalian cells (Wustner, 2007; Wüstner and Faergeman, 2008). This uniform cholesterol distribution is also supported by subsequently published super-resolution fluorescence imaging studies that showed fluorescent cholesterol analogs are not trapped in nanoscale domains within the plasma membranes of living cells (Hiramoto-Yamaki et al., 2014; Honigmann et al., 2014). Additionally, a comprehensive series of control experiments rigorously excluded the possibility that the lipid organizations imaged with high-resolution SIMS were artifacts of analysis. First, the imaging of fluorescent sphingolipids on fibroblast cells that had been metabolically labeled with fluorescent sphingosine showed that large sphingolipid domains were visible on the living cells, and the shapes, sizes, and positions of these fluorescent sphingolipid domains were not altered by glutaraldehyde fixation (Figures 2A–C; Frisz et al., 2013b). Thus, fixation did not induce sphingolipid clustering, and the lateral diffusion of lipids within the membrane during fixation did not disperse the sphingolipid domains that were present in the plasma membrane while the cells were alive. Next, experiments in which the rare stable isotope, ^13^C, was incorporated into all lipid species and imaged in parallel with ^15^N-sphingolipids confirmed that the cells' plasma membranes were intact. Importantly, lack of ^13^C-enrichment, which would signify an excess of all lipid species, at the ^15^N-enriched domains conclusively demonstrated that the local elevations in ^15^N-enrichment were not due to the detection of intracellular vesicles, organelles, or membrane folds, which would produce a co-committant increase (Figures 2D–F; Frisz et al., 2013b). Finally, control experiments ruled out the possibilities that the ^15^N-enriched domains on the cells were caused by isotope-labeled lipid precursors nonspecifically adsorbed to the cells, cell topography, temperature-induced domain formation, or sample preparation (Frisz et al., 2013b). Published reports have also established that high-resolution SIMS imaging does not alter the lipid distribution in phase-separated supported lipid bilayers (Kraft et al., 2006; Anderton et al., 2011), and this technique has the sensitivity to detect nanoscale domains enriched with GM1 and cholesterol in model lipid membranes (Lozano et al., 2013).

The lack of cholesterol enrichment in the sphingolipid domains detected on the fibroblast cells suggests that the self-organizing potential of cholesterol and sphingolipids is not responsible for plasma membrane organization. This possibility was further assessed by imaging the distributions of ^15^N-sphingolipids and ^18^O-cholesterol following cholesterol depletion. SEM images of mouse fibroblast cells that had been treated with methyl-β-cyclodextrin, which reduced the cellular cholesterol by 30%, showed cholesterol depletion altered cell morphology and reduced cell spreading. High-resolution SIMS imaging revealed the abundance of ^15^N-sphingolipid domains in the plasma membrane also decreased, but the remaining ^18^O-cholesterol in the plasma membrane still appeared to be relatively uniformly distributed (Frisz et al., 2013a). No significant difference in the ^18^O-cholesterol abundance in the sphingolipid domains and comparably sized non-domain regions was detected. Other mβCD-treated Clone 15 cells had similar cholesterol and sphingolipid distributions (Frisz et al., 2013a). Based on the lack of cholesterol enrichment in the sphingolipid-enriched domains either before or after cholesterol depletion, favorable cholesterol-sphingolipid interactions cannot be the driving force for plasma membrane organization.

**Figure 3:**
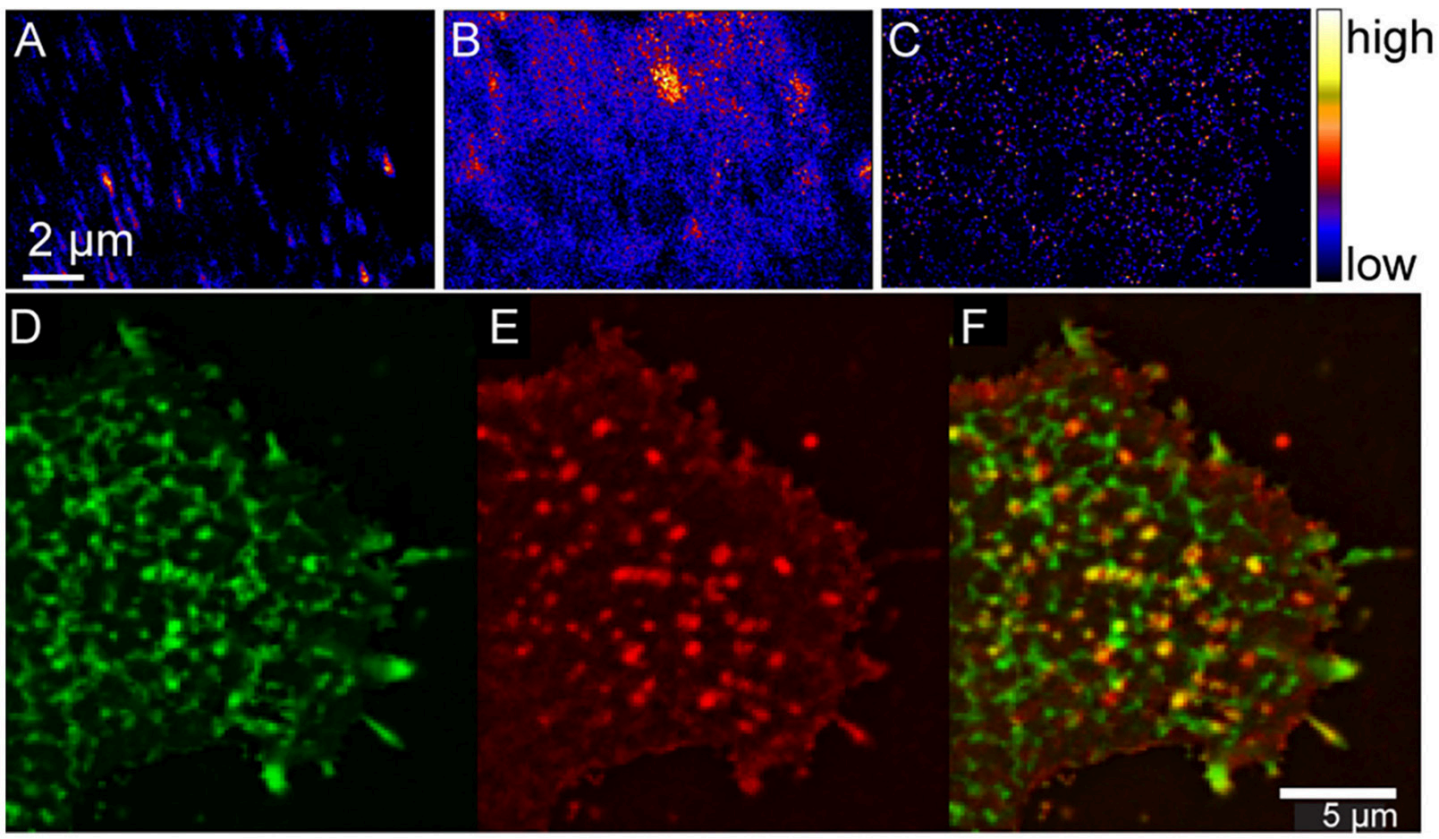
**High-resolution SIMS and complementary immunofluorescence imaging shows hemagglutinin does not cluster in plasma membrane domains that are enriched with cholesterol and sphingolipids**. High-resolution SIMS images of a region on a mouse fibroblast cell that stably expressed influenza hemagglutinin (Clone 15 cell line). **(A)** High-resolution SIMS image of the ^19^F^−^ counts shows the distribution of immunolabeled hemagglutinin in the plasma membrane. Comparison to the **(B)** ^15^N-enrichment and **(C)** ^18^O-enrichment images that were simultaneously acquired indicates hemagglutinin is not located in cholesterol- and sphingolipid-enriched domains. Reprinted from Wilson et al. (2015). Copyright (2015) with permission from Elsevier. Total internal reflectance microscopy detection of **(D)** BODIPY-sphingolipids (green) and **(E)** hemagglutinin (red) in the plasma membrane of a living Clone 15 cell. **(F)** Overlay shows little colocalization between the sphingolipids and hemagglutinin (yellow). Scale bar is 5 μm. Reproduced with permission from Frisz et al. (2013b). Copyright 2013 National Academy of Sciences, U.S.A.

The resemblance in the sphingolipid and cholesterol distributions in the plasma membranes of the Clone 15 and NIH 3T3 mouse fibroblast cells suggests the sphingolipid domains were not produced by favorable hemagglutinin-sphingolipid interactions. However, hemagglutinin might have an affinity for sphingolipids in the plasma membrane, which would cause the hemagglutinin to accumulate within the sphingolipid-enriched domains. This possibility was assessed by studying the stably expressed influenza hemagglutinin clusters in the membranes of uninfected Clone 15 cells instead of those in the membranes of influenza-infected cells to ensure that other viral proteins did not affect hemagglutinin localization within the plasma membrane. To permit visualization, the hemagglutinin on the metabolically labeled Clone 15 cells was labeled with a mouse anti-hemagglutinin antibody followed by an anti-mouse secondary antibody conjugated to a fluorinated colloidal gold particle (Wilson et al., 2012). High-resolution SIMS imaging of the ^19^F^−^ ions distinctive to the immunolabeled hemagglutinin in parallel with the ^15^N-sphingolipids and ^18^O-cholesterol revealed the fluorine-rich patches that located the hemagglutinin clusters were neither enriched with cholesterol nor well colocalized with ^15^N-sphingolipid domains (Figures 3A–C; Wilson et al., 2015). The low co-localization between the hemagglutinin and sphingolipid domains was confirmed by complementary experiments in which immunolabeled hemagglutinin and fluorescent sphingolipids in living Clone 15 cells were imaged with fluorescence microscopy (Figures 3D–F; Frisz et al., 2013b). The consistency between the findings of these two complementary techniques discounts the prospect that cell fixation or antibody labeling altered the membrane organizations observed with either technique. These findings disprove the hypothesis that hemagglutinin clustering is caused by an attraction to ordered plasma membrane domains that are enriched with cholesterol and sphingolipids. This conclusion is consistent with biophysical studies that indicated hemagglutinin is not located within cholesterol-rich liquid-ordered membrane domains (Hess et al., 2005, 2007; Polozov et al., 2008; Nikolaus et al., 2010).

**Figure 4:**
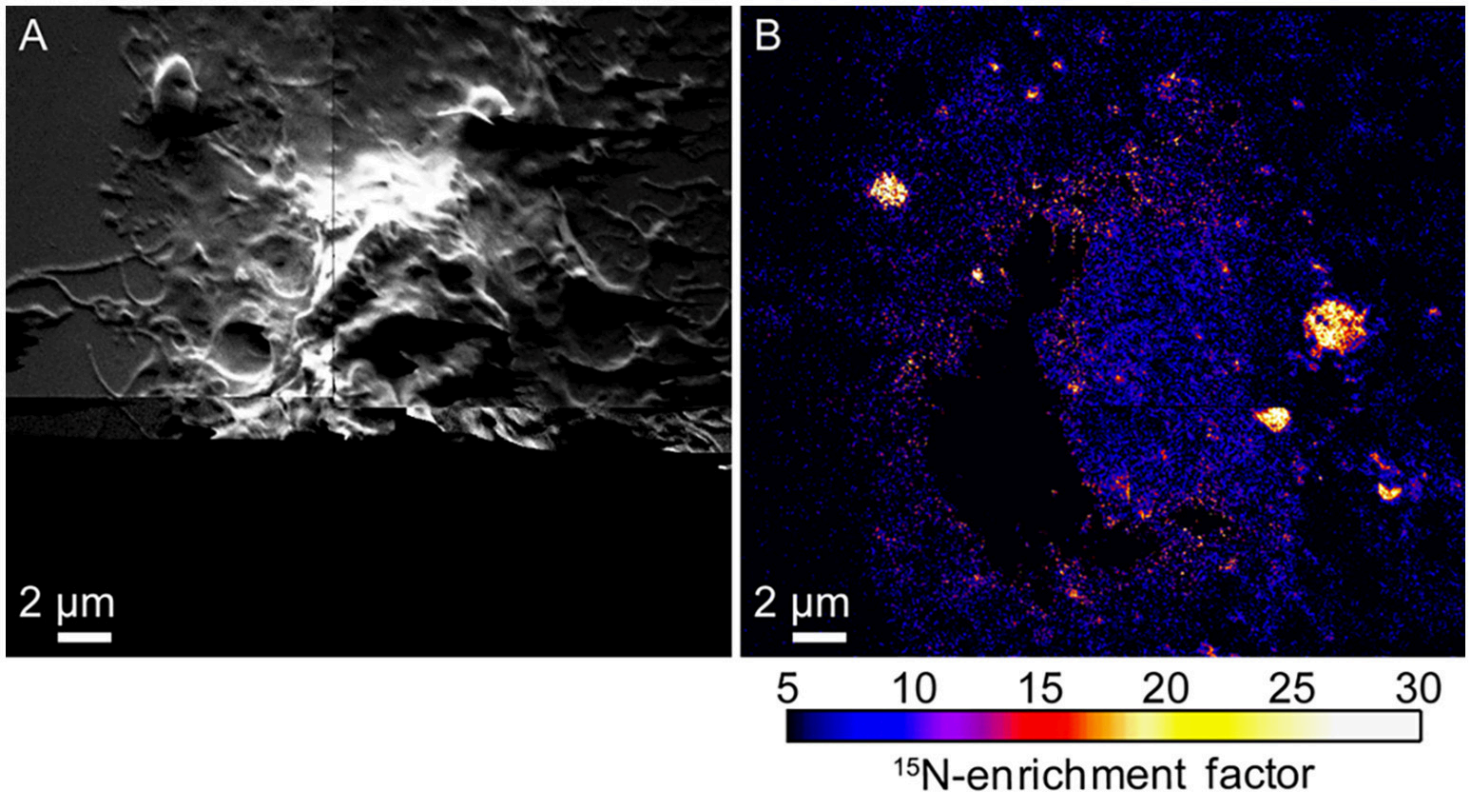
**Secondary electron and SIMS images of a NIH 3T3 fibroblast cell treated with latrunculin A to depolymerize the actin cytoskeleton. (A)** Secondary electron images show cell morphology. Secondary electrons were not detected at the bottom of the image due to the low beam current used. **(B)** ^15^N-enrichment images acquired with high-resolution SIMS show few ^15^N-sphingolipid domains following actin depolymerization. Color scales show the number of times that the ^15^N-enrichment is greater than standard abundance. Reproduced with permission from research originally published in Frisz et al. (2013a). © The American Society for Biochemistry and Molecular Biology.

The finding that cholesterol depletion reduced both cell spreading and sphingolipid domain abundance in the plasma membrane is consistent with the alternative hypothesis that the cytoskeleton and its associated proteins divide the plasma membrane into distinct lipid domains (Gheber and Edidin, 1999; Douglass and Vale, 2005; Kusumi et al., 2005; Hiramoto-Yamaki et al., 2014). This alternative hypothesis was also tested by using high-resolution SIMS to image the ^15^N-sphingolipid distributions in the plasma membranes of NIH 3T3 cells that were treated with latrunculin A to depolymerize their cytoskeletons. Actin depolymerization altered cell morphology (Figure 4A) and eliminated the vast majority of large ^15^N- sphingolipid domains in the plasma membrane (Figure 4B; Frisz et al., 2013b). This finding confirms the hypothesis that the cytoskeleton and its associated membrane proteins corral the sphingolipids within distinct domains in the plasma membrane.

## Implications for plasma membrane organization hypotheses

Independent experiments performed with complementary imaging techniques have yielded data that undeniably refutes the hypothesis that cohesive sphingolipid-cholesterol interactions are the driving force for plasma membrane organization. These findings include: (1) the lack of cholesterol- or hemagglutinin-enrichment in the sphingolipid domains that were detected in the plasma membranes of fibroblast cells with high-resolution SIMS (Frisz et al., 2013a; Wilson et al., 2015); (2) the unhindered diffusion of cholesterol analogs detected in the membranes of living cells with super-resolution fluorescence imaging (Hiramoto-Yamaki et al., 2014; Honigmann et al., 2014); and (3) the transient trapping of other putative raft components is inconsistent with interactions with rafts or lipid phase separation (Hiramoto-Yamaki et al., 2014; Honigmann et al., 2014; Sevcsik et al., 2015). Thus, although favorable cholesterol-sphingolipid interactions induce the formation of liquid-ordered domains that are enriched with cholesterol and sphingolipids in model membranes (Sankaram and Thompson, 1990) and membrane blebs (Baumgart et al., 2003, 2007), these interactions do not control lipid organization in the plasma membranes of actual cells. Given that cholesterol-sphingolipid interactions are a cornerstone of the lipid raft hypothesis and both high-resolution SIMS and super-resolution fluorescence techniques failed to detect lipid rafts, these results not only argue against the existence of rafts, they conclusively disprove their existence.

The discrepancies between experimental data and predictions of the raft hypothesis cannot be rectified by incorporating additional protein-protein or protein-lipid interactions into a revised model that is still based on cohesive sphingolipid-cholesterol interactions. Instead, alternative hypotheses that do not involve cohesive sphingolipid-cholesterol interactions must be developed, investigated, and discarded if they prove inconsistent with experimental results. These alternative hypotheses should account for the following observations:

- The diffusion and distribution of proteins and lipids is influenced by the actin cytoskeleton (Fujiwara et al., 2002, 2016; Mueller et al., 2011; D'Auria et al., 2013; Frisz et al., 2013a,b; Honigmann et al., 2014; Andrade et al., 2015; Sevcsik et al., 2015; Komura et al., 2016).
- Actin accumulates under clusters of crosslinked membrane proteins (Ash et al., 1977; Bourguignon and Singer, 1977; Kellie et al., 1983; Pierini et al., 1996; Harder and Simons, 1999; Rodgers and Zavzavadjian, 2001; Delaguillaumie et al., 2004; Wilson et al., 2004; Goswami et al., 2008; Gowrishankar et al., 2012; Gudheti et al., 2013).
- Different sphingolipid subspecies form separate microdomains in the plasma membrane, and each domain of different sphingolipid subspecies may contain distinctly different membrane proteins (Fujita et al., 2007, 2009; Janich and Corbeil, 2007; Chen et al., 2008; Tyteca et al., 2010).
- Cellular processes are sensitive to sphingolipid catabolism and inhibitors of sphingolipid biosynthesis (Wiegmann et al., 1994; Tepper et al., 1995; Grassmé et al., 1997, 2001a,b, 2003a; Brenner et al., 1998; Junge et al., 1999; Grullich et al., 2000; Cremesti et al., 2001; Paris et al., 2001; Grassmé et al., 2003b; Abdel Shakor et al., 2004, 2012; Grassmé, 2005; Korzeniowski et al., 2007; Verdurmen et al., 2010; Serrano et al., 2012).
- Cholesterol depletion affects protein clustering and cell signaling (Stauffer and Meyer, 1997; Harder et al., 1998; Harder and Simons, 1999; Huby et al., 1999; Janes et al., 1999; Cremesti et al., 2001; Grassmé et al., 2001a; Mitchell et al., 2002; Lacour et al., 2004; Hess et al., 2005).

The alternative hypothesis that the plasma membrane is segregated by cortical actin and its associated proteins is consistent with the numerous observations that the distribution and diffusion of lipids and proteins in the plasma membrane is influenced by drugs that affect cytoskeletal integrity (Kusumi and Sako, 1996; Ritchie et al., 2003; Kusumi et al., 2005). In this model, the cytoskeleton and its associated proteins establish diffusion barriers, and the energy-dependent constant delivery and removal of membrane proteins and lipids at the plasma membrane creates lateral variations in component abundance (Gheber and Edidin, 1999; Turner et al., 2005; Lavi et al., 2007; Fan et al., 2010). Indeed, localized trafficking hubs in the plasma membrane have been shown to produce stable domains of distinct protein compositions (Deutsch et al., 2012; Fox et al., 2013). Whether the sphingolipid domains in the plasma membrane are local hubs for sphingolipid trafficking might be assessed by performing high-resolution SIMS in a depth profiling mode to produce three-dimensional images of the intracellular sphingolipid distribution (Yeager et al., 2016).

Nonetheless, the true mechanism for plasma membrane organization is probably far more complex than the current cytoskeleton-based model. For example, cytoskeletal barriers combined with endocytosis and exocytosis events may not fully explain the reported redistribution of crosslinked gangliosides within the plasma membrane during capping. Therefore, the previous hypothesis that individual sphingolipid species selectively and reversibly interact with distinct proteins that are associated with the actin cortex may need to be reconsidered. These sphingolipid-protein interactions may be transient, regulated by external stimuli (i.e., ligand binding), and specific, where different sphingolipid subspecies bind to different protein partners. Such specific, inducible, and transient sphingolipid-protein interactions could direct the segregation of different glycosphingolipid species within different microdomains in the plasma membrane (Fujita et al., 2007, 2009; Janich and Corbeil, 2007; Chen et al., 2008), and mediate their clustering in response to crosslinking. This hypothetical mechanism may also account for colocalization between specific glycosphingolipid species and distinct proteins in the plasma membrane (D'Auria et al., 2013), and the accumulation of actin observed beneath clusters of membrane proteins (Ash et al., 1977; Bourguignon and Singer, 1977; Kellie et al., 1983; Pierini et al., 1996; Harder and Simons, 1999; Rodgers and Zavzavadjian, 2001; Delaguillaumie et al., 2004; Wilson et al., 2004; Goswami et al., 2008; Gowrishankar et al., 2012; Gudheti et al., 2013). Given the existence of lipid binding proteins that selectively interact with phosphatidylinositols, phosphatidylcholines, and phosphatidylserines (Lemmon, 2008; Stahelin, 2009; Glatz, 2015), other lipid species may also selectively bind to distinctive proteins that are associated with the actin cortex.

The sensitivity of many cellular processes, including antigen capping and apoptosis, to enzymes that induce sphingolipid catabolism or drugs that inhibit sphingolipid biosynthesis can be attributed to the established role of sphingolipids and their metabolites as second messengers in diverse signaling processes (Hannun and Obeid, 2008; Zeidan et al., 2008; Kim et al., 2009; Milhas et al., 2010; Spiegel and Milstien, 2011; Canals et al., 2012). The cholesterol sensitivity of membrane protein clustering and other events that occur in the plasma membrane may be indicative of specific cholesterol-protein interactions (Lange and Steck, 2016). Cholesterol is known to selectively bind to specific sites on a few integral membrane proteins, thereby regulating their conformation and activity (Hanson et al., 2008; Fürst et al., 2014; Clay et al., 2015). The observation that cholesterol depletion reduces cell spreading may suggest that cholesterol binding regulates plasma membrane attachment to the cytoskeleton. Alternatively, cholesterol may indirectly affect membrane attachment to the cytoskeleton via its effects on the abundance of phosphoinositides in the plasma membrane, which help to recruit cytosolic proteins to the plasma membrane (Kwik et al., 2003). A combination of affinity labeling, mass spectrometry detection of protein complexes associated with distinct lipids or cholesterol, and super-resolution imaging of suspected binding partners in cells will be required to evaluate this hypothesis.

## Author contributions

MK created figures and wrote the paper.

### Conflict of interest statement

The author declares that the research was conducted in the absence of any commercial or financial relationships that could be construed as a potential conflict of interest.
